# Presence and genetic variability of *Staphylococcus aureus* subsp. *anaerobius* isolated from small ruminants in Central Europe

**DOI:** 10.1038/s41598-025-97300-z

**Published:** 2025-04-18

**Authors:** Benjamin Ulrich Bauer, Christina Ambros, Kerstin Boll, Johanna Hilke, Julia Magarete Riehm, Olga Szaluś-Jordanow, Cornelia Schneider, Eva Sodoma, Thomas Bauz, Martin Ganter, Sven Maurischat

**Affiliations:** 1 Clinic for Swine and Small Ruminants, Forensic Medicine and Ambulatory Service, University of Veterinary Medicine Hannover, Foundation, Bischofsholer Damm 15, 30173 Hannover, Germany; 2 Institute of Immunology, Friedrich-Loeffler-Institut, Südufer 10, 17493 Greifswald, Isle of Riems, Germany; 3 Bavarian Animal Health Service, Senator-Gerauer-Str. 23, 85586 Poing, Germany; 4 Bavarian Health and Food Safety Authority, Veterinärstraße 2, 85764 Oberschleißheim, Germany; 5 Schafpraxis, Am Hopfenberg 8, 89352 Stoffenried-Ellzee, Germany; 6 Department of Small Animal Diseases with Clinic, Institute of Veterinary Medicine, Warsaw University of Life Sciences-SGGW, Nowoursynowska 159c, 02-776 Warsaw, Poland; 7 Austrian Agency for Health and Food Safety GmbH (AGES), Spargelfeldstraße 191, 1220 Vienna, Austria; 8 Tierarztpraxis Bauz, Tetta 21b, 02894 Vierkirchen, Germany; 9 Department Biological Safety, National Reference Laboratory for coagulase-positive staphylococci including Staphylococcus aureus, German Federal Institute for Risk Assessment (BfR), Max-Dohrn-Str. 8-10, 10589 Berlin, Germany

**Keywords:** Antimicrobial resistance, Goat, Morel’s disease, Multilocus sequence typing, Sheep, Virulence factor, Microbiology, Diseases

## Abstract

*Staphylococcus aureus* subsp. *anaerobius* (SAAN) causes abscesses in small ruminants, known as Morel’s Disease. This study describes the presence of SAAN for the first time in Germany and Austria and examines the phylogenetic relationship among these isolates and previously described European and Sudanese ones. A total of 35 sheep and 10 goat isolates from 12 herds in Germany were available for analysis. SAAN isolates from four Polish goats and three Austrian sheep from different herds were included. Genome comparisons and phylogenetic analyses were conducted using core genome multilocus sequence typing. The comparison of the 52 SAAN core genomes revealed a close phylogenetic relationship among most German isolates (*n* = 38), with allelic differences ≤ 6 in two clusters associated with ST4581. In contrast, distinct clusters of the same ST included the four Polish goat isolates and two ovine isolates from Austria, respectively. A fifth cluster of ST3756 strains was identified on three German farms (six sheep, one goat) and an Austrian sheep. Tight phylogenetic relationships were observed irrespective of the host species. All isolates shared a common set of virulence genes and few known antimicrobial resistance determinants. The introduction of SAAN into herds is mostly unknown, but purchases appear to play a critical role.

**Supplementary Information:**

The online version contains supplementary material available at 10.1038/s41598-025-97300-z.

## Introduction

The bacterium *Staphylococcus (S.) aureus* subsp. *anaerobius* (SAAN) was first described in France by Morel in 1911^1^. Bacteriological identification of SAAN is delicate; it typically does not sprout under aerobic conditions but grows slowly in microaerophilic or anaerobic atmospheres. SAAN causes abscesses, primarily in close association with superficial lymph nodes in sheep and goats^2,3^. This disease is known as Morel’s Disease (MD) or abscess disease and has been reported in small ruminant populations in many African and Middle Eastern countries^4–9^. Since 2000, reports of MD in small ruminants have increased throughout Europe, including countries such as Denmark^2^, Croatia^10^, Poland^3^, Switzerland^11^, and the Czech Republic^12^. In Spain, SAAN was even identified as the most frequent causative agent of abscesses in small ruminants^13^. To date, SAAN has not been reported in sheep and goats from Germany and Austria^14^.

MD is endemic and contagious and is mainly encountered in young animals within the first 4–10 months of life^15^. However, adult animals are also severely affected, especially when the pathogen enters a herd for the first time^3^. Transmission occurs through direct or indirect contact with pus from spontaneously drained abscesses. International trade with sheep and goats is a significant risk factor for introducing SAAN into naïve herds^2,3,12^. MD causes financial losses due to rejection of shipments or condemnation of sheep carcasses during meat inspection in slaughterhouses. Nevertheless, the exact economic losses due to MD have not yet been computed^16^. The zoonotic potential of SAAN has been suspected, but final proof thereof is still pending^17^.

Recently, a detailed study of the SAAN genome revealed a highly conserved clone, tracing its lineage back to a *Staphylococcus (S.) aureus* progenitor that existed approximately 1,000 years ago^18^. The biochemical identification of SAAN is very limited due to lack of growth under aerobic conditions. Additionally, commercially available kits, such as API 20 A, API rapid ID 32 A, or VITEK ANI card (all BioMerieux, Nuertingen, Germany) do not contain anaerobic staphylococci in their databases^17^. In biochemical identification algorithms, the presence of catalase is listed as a characteristic of *S. aureus*^19^. This virulence factor degrades hydrogen peroxide to water and oxygen. Produced in vivo by bacteria, the enzyme prevents chemical damage, for example, after phagocytosis by neutrophils. However, SAAN is a catalase negative organism, and its virulence potential should not be underestimated. It is assumed that this microaerophilic metabolism is because of the loss of catalase function and other oxidoreductase genes, resulting in higher susceptibility to oxidative free radicals^18^. In contrast to the sometimes misleading results of microbiological or biochemical approaches, a reliable identification at species level is possible using the molecular approach based on the *16S*rRNA gene sequence analysis^17^ or by MALDI-TOF analysis. A further differentiation below species level can be realized by different molecular typing tools. Genetic analyses of SAAN strains from several countries using different methods such as pulsed-field gel electrophoresis (PFGE), multilocus sequence typing (MLST), and random amplified polymorphic DNA (RAPD) have revealed very high homogeneity among these isolates^14,20^. Nevertheless, two distinct lineages of SAAN containing African and European isolates exist^18^.

Interest has been steadily growing in the remarkably high number of toxins and other virulence factors produced by *S. aureus* and their impact on disease, as recently reviewed by Cheung and colleagues^21^. For SAAN, the *vwb* gene, encoded on one of the two mobile genetic elements (MGEs) in the accessory genome, is considered highly relevant for forming classical abscesses in MD^18^. Moreover, antimicrobial resistance (AMR) is very prominent in many *S. aureus* strains, especially those that pose a major health threat in humans and animals, like methicillin-resistant *S. aureus*, and may be conferred by gene modifications or resistance genes that are encoded chromosomally or on MGE^22^. In contrast, AMR determinants in SAAN are still largely unknown.

Due to the emerging numbers of SAAN isolation in small ruminant herds in Germany, a study was performed to describe the presence of MD, to characterize them, and to examine the phylogenetic relationship among SAAN isolates from different German regions. In addition, samples from Poland and Austria as well as genomic sequences from public databases were included in the genetic analysis to investigate a possible connection. To obtain a complete picture of the Central European SAAN isolates, virulence factors and AMR determinants were assessed. Furthermore, the zoonotic potential of SAAN was discussed due to carcass condemnations caused by abscesses in a significant number of lambs within the herds presented here.

## Material and methods

**Fig. 1:**
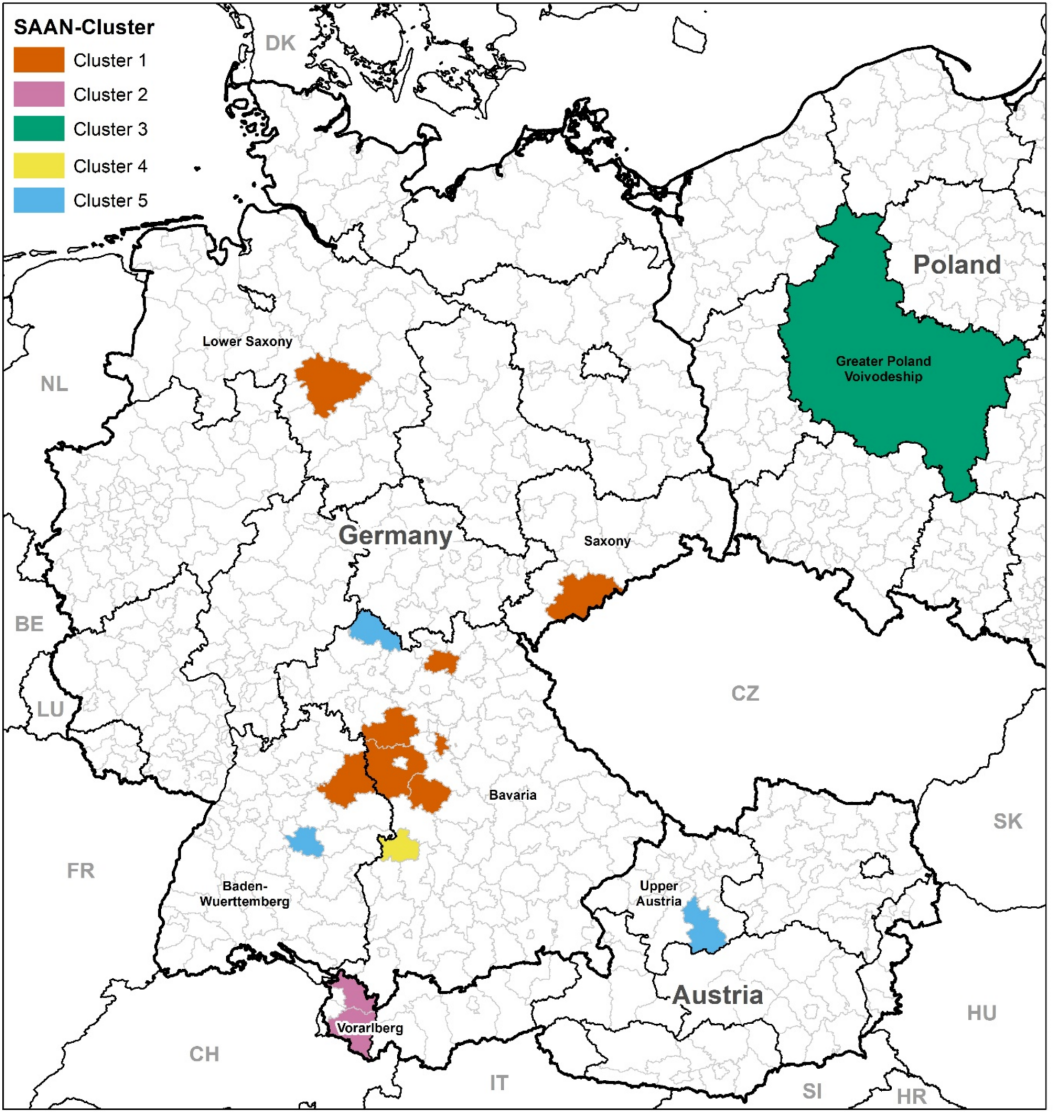
Location of the *Staphylococcus aureus* subsp. *anaerobius* (SAAN)-positive sheep and goat herds in Germany, Austria, and Poland. Different colors indicate different genomic clusters of SAAN based on cgMLST analysis. © GeoBasis-DE / BKG (2023).

Samples were collected from sheep and goats from 17 small ruminant farms, with 12 herds located in Germany, three sheep herds in Austria, and two dairy goat herds in Poland (Fig. 1). In total, 35 sheep and 10 goats were sampled between December 2018 and September 2022 in Germany. These animals had developed abscesses in the region of the superficial lymph nodes and were sampled during controlled abscess lancing by the authors. Moreover, the farmers were concerned about carcass condemnations at the slaughterhouse due to abscesses in several lambs, which seemed to increase their willingness to engage with local veterinarians to identify the cause of the disease. In addition to the German specimens, four samples from two Polish dairy goat farms, obtained from a previous study in 2006–2007^3^ were included, along with three samples from Austrian sheep obtained from routine diagnostics. Detailed information about the analyzed samples is given in Table 1 and in Supplementary Table 1.

The study was approved as it complied with the animal welfare guidelines of the University of Veterinary Medicine Hannover, the German Animal Welfare Act, and Directive 2010/63/EU of the European Parliament and of the Council of 22 September 2010 on the protection of animals used for scientific purposes. All animal procedures were reviewed and approved by the Ethics Committee of the University of Veterinary Medicine Hannover (approval code: TiHo_EA_14_24–24) and were conducted in accordance with the ARRIVE guidelines. Animal-derived data were obtained exclusively for diagnostic purposes. Consequently, the use of these data did not require additional authorization from the IACUC or the competent authority under Directive 2010/63/EU.

**Table 1:** Details about *Staphylococcus aureus* subsp. *anaerobius* (SAAN) isolates detected in abscess material from sheep and goats in Germany, Austria, and Poland.

Farm/ Sample ID	Species	Location of the Herd (Country, State, District)	Potential Source of SAAN	ST
G1.1-G1.10	Sheep	DE, Bavaria, Lichtenfels	Purchase of sheep	4581
G4	Goat	DE, Lower Saxony, Hanover	Unknown	
G5.1- G5.4	Sheep	DE, Bavaria, Dillingen	Unknown	
G6.1- G6.4	Sheep	DE, Baden-Wuerttemberg, Schwaebisch Hall	Unknown	
G6.5	Goat			
G7.1- G7.4	Sheep	DE, Bavaria, Neustadt/Aisch	Purchase from G1	
G9.1- G9.3	Goat	DE, Bavaria, Nuremberg	Unknown	
G10	Goat	DE, Bavaria, Ansbach	n/a	
G11.1- G11.4	Sheep	DE, Bavaria, Weissenburg-Gunzenhausen	Unknown	
G11.5	Goat			
G12.1-G12.3	Sheep	DE, Saxony, Erzgebirgskreis	Purchase of sheep	
G12.4- G12.5	Goat			
A2	Sheep	AT, Vorarlberg, Bregenz	Purchase from France	
A3	Sheep	AT, Vorarlberg, Bludenz	n/a	
P1.1- P1.3	Goat	PL, Greater Poland Voivodeship	Buck from DE	
P2	Goat		Purchase from P1	
G2.1-G2.2	Sheep	DE, Bavaria, Rhoen-Grabfeld	Unknown	3756
G3.1-3.4	Sheep		Unknown	
G8	Goat	DE, Baden-Wuerttemberg, Esslingen	Purchase of goats	
A1	Sheep	AT, Upper Austria, Kirchdorf	n/a	

DE = Germany, AT = Austria, PL = Poland, n/a = not available, SAAN = *Staphylococcus aureu*s subsp. *anaerobius*, ST = multilocus sequence type.

### Isolation of SAAN

The abscess samples were examined applying classic cultural bacteriologic methods. In brief, swabs were plated onto Columbia Agar with 5% sheep blood and incubated under aerobic, microaerobic, and anaerobic conditions for 24, 48, and 72 h at 37 °C. No growth was observed on all plates after 24 h. After 48 h, small white colonies were visible on plates incubated under microaerobic and anaerobic conditions, while there was still no growth on aerobic plates. Presumptive *S. aureus* colonies were subcultured and confirmed by MALDI-TOF MS. Apart from different ambient conditions, SAAN can be differentiated from *S. aureus* subsp. *aureus* by a negative catalase test.

### Whole-genome sequencing and bioinformatics analyses

Presumptive SAAN isolates and the DSM 20714 type strain were inoculated in 5 mL brain heart infusion broth and anaerobically incubated at 37 °C for 24 h. DNA of 1 mL culture was extracted using the Qiagen DNeasy Blood and Tissue Kit (Qiagen GmbH, Hilden, Germany) according to the manufacturer’s protocol, modified by adding 10 µL lysostaphin (0.1 mg/mL; Sigma-Aldrich Chemie GmbH, Taufkirchen, Germany) to the lysis buffer.

The DNA library was prepared using an Illumina Nextera DNA Prep kit (Illumina Inc., San Diego, CA, USA), and the 150 bp paired-end sequencing run was performed on an Illumina NextSeq 500 instrument. Raw Illumina reads were trimmed and de novo assembled using a SPAdes algorithm with the in-house developed AQUAMIS pipeline^23^. Bacterial characterization was conducted with the in-house developed BakCharak pipeline (https://gitlab.com/bfr_bioinformatics/bakcharak, accessed on February 8, 2023) using the NCBI AMRfinder database^24^ for screening of AMR genes, the virulence factor database (VFDB) SetB (http://www.mgc.ac.cn/VFs/, accessed August 26, 2022) for the detection of virulence genes, and MLST based on the scheme of Enright and colleagues^25^. For the presence of virulence and resistance genes, a 50% sequence coverage and 50% identity were considered. Genome comparisons and phylogenetic analyses were conducted by core genome multilocus sequence typing (cgMLST) using Ridom SeqSphere + version 7.0.4 (Ridom GmbH, Münster, Germany), and the integrated 1861 loci scheme was used^26^.

Additionally, raw paired-end reads of six publicly available genomes at the European Nucleotide Archive (ENA) from published strains^18^ that were isolated in Denmark, Italy, Poland, Spain, and the Sudan were assembled as described above and included in the cgMLST comparison. The respective strain IDs were complemented with ‘*_ENA’ and are listed in Supplementary Table 2.

## Results

### Phylogenetic analysis

**Fig. 2:**
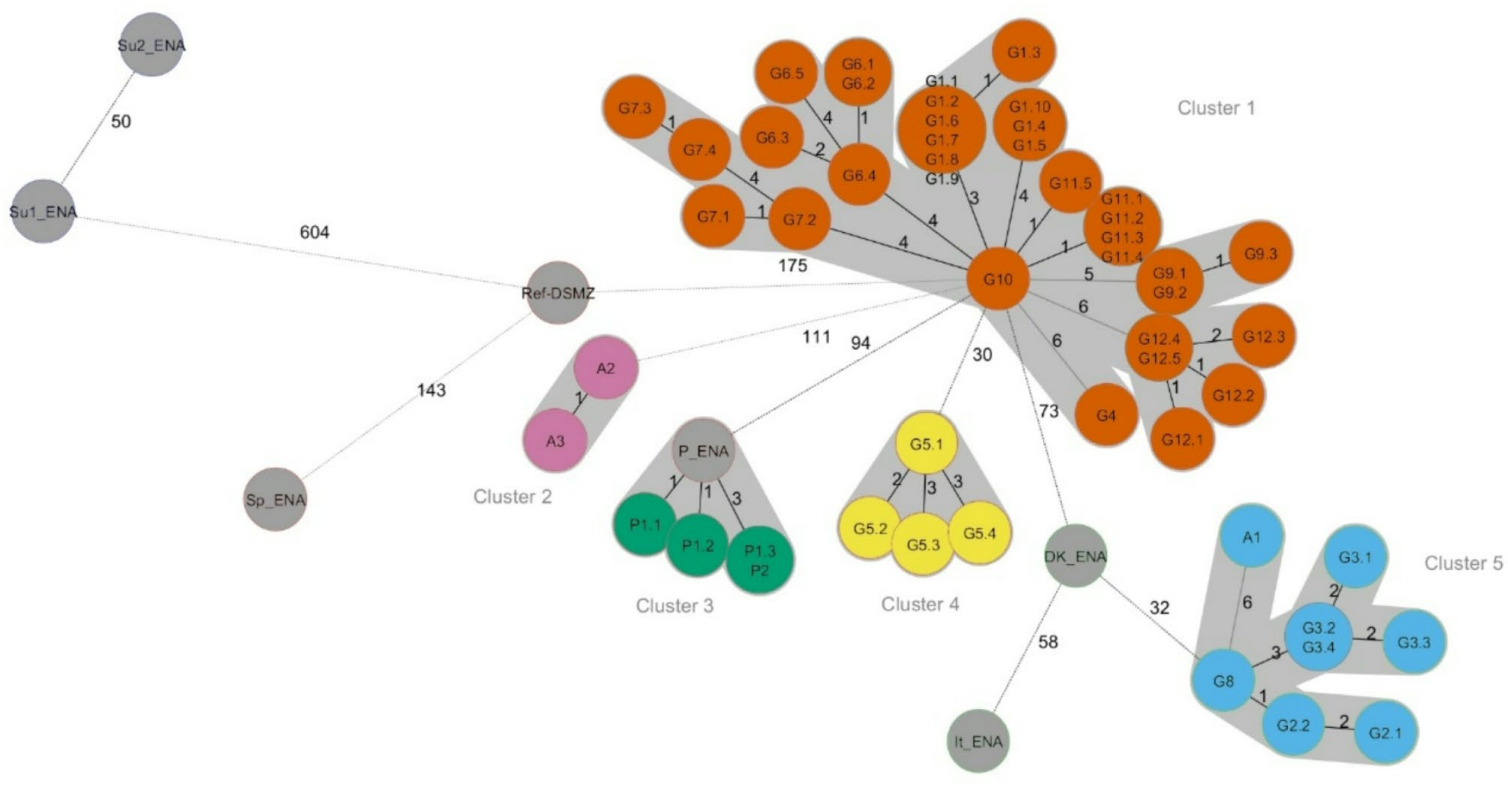
Minimum spanning tree based on comparative cgMLST analyses of SAAN isolates from this study, the type strain DSM 20714 and published genome sequences from the ENA database. The ENA strains and the DSM strain are marked in gray, whereas the isolates of this study are colored according to clusters 1–5. The allelic differences (AD) between strains are indicated as numbers between the nodes. The branch length is not proportionate to the allelic differences. Clusters are defined by strains with less than 10 ADs.

The comparison of the 52 SAAN core genomes from this study, the core genome of DSM 20714 (Ref-DSMZ), and those from the further international SAAN strains available at the ENA database revealed a close phylogenetic relationship of most German isolates (*n* = 34) with allelic differences (AD) ≤ 6 in Cluster 1 (Fig. 2). In contrast, we found further distinct clusters of the same ST4581 with the goat isolates and ENA strain from Poland (P1, P2, and P_ENA, Cluster 3), sheep isolates (*n* = 4) from Bavaria (G5, Cluster 4), two sheep isolates from Austria (A2, A3, Cluster 2), and singletons like the Ref-DSMZ and the Spanish ENA strain that were more distantly related. Furthermore, we identified a fifth cluster of ST3756 strains that were assigned to two Bavarian farms (G2, G3), one farm in Baden-Wuerttemberg (G8), and a sheep farm from Austria (A1) as well as two singletons with the ENA strains from the Sudan that belonged to ST5843.

Interestingly, the four isolates from Poland (Cluster 3) and two isolates from Austria (Cluster 2) each assigned to ST4581 were phylogenetically highly related with AD ≤ 4, whereas a relationship between isolates of different countries could only be assumed in the case of the German and Austrian ST3756 isolates (G8, A1, Cluster 5), with the caveat that a clustering cut-off in that case was hard to define due to the lack of definitively epidemiologically unrelated ST3756 strains.

Furthermore, tight phylogenetic relationships were observed irrespective of the host species (sheep, goat) from which they originated, indicating no association between the sequence type and a certain host species.

### Genotypic characterization: virulence-associated genes

The highly clonal structure and conserved genome could also be confirmed by analyzing genes that are associated with virulence and antimicrobial resistance. All isolates from this study independent of their ST harbored the same classical *S. aureus* virulence genes with relevance for adhesion like *cna* (collagen binding), *eap* (extracellular adherence protein), *emp* (extracellular matrix protein), *cflB*,* efb* and *fnbA* (fibronectin-binding), *sas* (*S. aureus* surface protein), *srtB* (sortase B), the expression of a type VII secretion system like the *esa/ess/esx*-operon, and the production of exotoxins (hemolysins: *hlb*,* hld*,* hlgB*,* hlgC*,* hly;* staphylococcal superantigen-like proteins: *set25*,* set30*,* set31;* leukotoxins: *lukD*,* lukE*,* lukG*,* lukH*) as well as exoenzymes (aureolysin: *aur*; hyaluronate lyase: *hysA*; lipase: *geh*, *lip*; coagulase: *coa*; serine proteases: ssp.,* spl*; Von Willebrand factor-binding protein (vWbp): *vwb*). Furthermore, genes that affect immune evasion, like *adsA* (Adenosine synthase A), capsule-related genes (*cap8B*,* cap8C*,* cap8E*,* cap8F*,* cap8H* to *cap8M*,* cap8O*,* cap8P*,* capA*,* capN*), and *sbi* (staphylococcal binder of immunoglobulin), or are important for biofilm synthesis like the *ica*-operon, the *sdrC* gene, and the autolysin gene *atl* were present in all genomes. Iron acquisition is crucial for host infection and associated gene operons like *sbn*, *sfa*, and *sir* could be identified. All of these virulence genes were chromosomally encoded.

### Genotypic characterization: antimicrobial resistance genes

The number of AMR associated genes or genetic modifications in SAAN were very limited and the same for all isolates in this study. We identified point mutations in the genes *glpT*_A100V, *murA*_E291D, and *murA*_T396N that are associated with fosfomycin resistance, and detected the *mepA* and *tet(38)* genes which confer resistance to tetracyclines. Furthermore, the *lmrS* gene of the lincomycin resistance protein, an efflux pump that belongs to the major facilitator superfamily^27^, was present. All genes were encoded chromosomally, plasmids could not be identified.

## Discussion

To the best of the authors’ knowledge, this study describes the presence of SAAN in small ruminants located in Germany and Austria for the first time. However, it is noteworthy that a German breeding buck potentially introduced SAAN to a naïve Polish dairy goat herd in 2005^3^. Therefore, MD appears to have been present in the German small ruminant population before. The reason for the sudden emergence in sheep herds in southern Germany is still unclear and might be associated with increased awareness of farmers and veterinarians. Besides the already described likely transmission from a German buck to a Polish goat herd, two SAAN outbreaks were reported in Denmark^2^ and the Czech Republic^12^, caused by the importation of sheep from France. However, current information about the presence of SAAN in France exists only for goat herds^28^. This emphasizes the need to implement quarantine measures, such as isolation and palpation of animals’ superficial lymph nodes, even for small ruminants from countries where SAAN has not yet been reported. In the present study, limited information was available regarding the source of SAAN introduction to small ruminant herds, though some farmers suggested that animal purchases may be responsible. Additionally, shearing has been reported as a risk factor for SAAN transmission to naïve herds^15^. Only one shearing contractor (Contractor A) sheared three of the sheep flocks included in this study (flocks G1, G3, and G7; see Supplementary Table 1), while other flocks were sheared by different contractors. However, SAAN transmission from flock G1 to flock G3 appears unlikely, as different MLST types were detected in the two flocks. Furthermore, SAAN was introduced to flock G7 due to the purchase of lambs from flock G1. Thus, shearing seems to play a less significant role in the transmission of SAAN among the herds studied here, but the small sample size limits the epidemiological traceability of SAAN transmission among sheep farms.

Interestingly, even though herds G2 and G3 were located in the same district and belong to the same cluster, we can exclude any animal movements between both farms, as they are pedigree breeders of different sheep breeds, and cross-breeding did not occur. Although the owners of herds G2 and G3 hired different shearing contractors (Supplementary Table 1), an indirect contact, e.g., by vehicles, cannot be completely ruled out. It is known that *S. aureus* can survive for up to seven months on inanimate surfaces^29^, but information about the longevity of SAAN is lacking. Other anaerobic bacteria, such as *Bacteroides melaninogenicus* and *Fusobacteria nucleatum*, can tolerate air for up to 72 h^30^. Such a short oxygen tolerance has to be proven for SAAN in the future. Moreover, ticks, in combination with the tick-borne bacteria *Anaplasma (A.) phagocytophilum*, favor tick pyemia caused by *S. aureus* in lambs^31^. This combination of tick infestation and *A. phagocytophilum* might also trigger the onset of clinical MD cases, as *A. phagocytophilum* is very common in the German sheep population and the pathogen has an immune-suppressive effect on small ruminants^32^.

We have described the relationship of isolates from different herds within Germany and strains from neighboring and distant countries, which allows us to draw conclusions regarding potential transmission pathways. However, we have not found any clear indications of an international transmission of strains within the last years. The isolates analyzed in this study mostly revealed a distant relationship across country borders, but the conclusion is limited due to the lack of complete surveillance data. Moreover, in the case of potential introduction into a Polish dairy goat herd, the time difference between the sampling of the goat herd and the German small ruminant herds is approximately 12 years, which additionally hampers the comparison. Nevertheless, single transmission events cannot be excluded and could be exemplarily present in the relationship of isolates G8 and A1 as part of Cluster 2, with the limitation of an uncertain cut-off between related and unrelated ST3756 strains. In addition, the same strain was also identified in sheep from the Czech Republic^12^, but a French origin of SAAN was suspected, which indicates a pathogen transmission among four countries. However, transmission events between herds and regions in Germany are more frequent than between countries, which results in the formation of endemic SAAN populations that can be phylogenetically distinguished using highly discriminatory molecular methods like single nucleotide polymorphism or cgMLST analyses.

Most of the virulence genes detected in the SAAN isolates occur ubiquitously in *S. aureus*. However, Yebra et al.^18^ described the frequent occurrence of pseudogenes in SAAN isolates with approximately 8% of the genome in contrast to *S. aureus* subsp. *aureus* isolates due to point mutations that affect the transcription of these genes. This indicates that beyond the pure detection of virulence and resistance genes in our study, statements regarding their functionality are very limited. Insofar some of the above-mentioned virulence genes might be probably inactive, namely *capH8*,* cflB*,* emp*,* fnbA*,* hly*,* lip*,* lukD/E*, and *sbi* which were described as pseudogenes and seem not relevant or beneficial regarding the adaptation to ruminants. In contrast, others do not show signs of impairment so far and are also reported to be relevant for attachment (*cna*,* eap*,* efb*,* sas*,* srtB*) or cytotoxicity (*hlb*,* hld*,* hlgB*,* hlgC*). Especially those encoding the Type VII Secretion System (*esa/ess/esx*-operon)^33^, which is important for pathogenicity by secreting toxins and other virulence factors as well as the interbacterial competition do not include pseudogenes. Further virulence genes that are similarly generalist and ensure the iron acquisition (*sbn*,* sfa*, *sir*), or confer advantages in the environment by enabling the production of biofilms (*atl*,* ica*, *sdrC*) have not been evolutionary pseudogenized.

The results reported here and elsewhere have shown the fundamental reduction of virulence genes in contrast to *S. aureus* subsp. *aureus*. On the other hand, a few virulence genes are much more prevalent in SAAN than in *S. aureus* subsp. *aureus* isolates like *vwb* or *cna*. Coagulase production is a prerequisite for coagulase-positive Staphylococci like *S. aureus*, but in contrast to *coa*, which is the classical mediator in virtually all *S. aureus* strains, *vwb* is only present in certain *S. aureus* strains. Both factors have been proven to synergistically affect the abscess formation in mice^34^ although each protein is able to coagulate plasma. The presence of the *vwb* gene in contrast to *coa* is, according to Pickering and colleagues^35^, the result of a host-dependent evolution, with a host-specific functionality especially with regard to ruminants and equines^36^. This was concurrently confirmed for SAAN by Yebra and colleagues^18^ who highlighted vWbp as an important virulence factor, which is encoded on one of only two MGEs in the accessory genome of SAAN strain MVF7, the SAAN pathogenicity island SaaPIMVF7, and hypothesized this particular *vwb* variant to be highly relevant for the formation of classical abscesses in MD^18^.

Another example of a gene which was prevalent in all SAAN isolates in this study but not very common in other *S. aureus* is *cna*, which encodes an adhesin that enables the binding to collagen^37^ and thereby resists clearance by the host defense mechanisms. It was observed that *cna* is especially prevalent in certain *S. aureus* clonal complexes (CC) like CC1, 12, 22, 30, 45, 51, and 239^38^, and that this might be linked to a colonizing rather than an invasive lifestyle. Furthermore, this gene was recently reported to be prevalent in atypical CC133 *S. aureus* from waterfowl^39^. However, it is yet not clear what drives this uneven distribution of *cna* between different *S. aureus* types and how far it could be linked to the adaptation to ruminants in SAAN or the progress of MD. Furthermore, it must be constrained that the *cna* reference sequence in the VFDB Set B was only covered by 61% by the assembled SAAN sequences. Thus, it is not yet ruled out that this gene is not functional in SAAN or is just a pseudogene.

Similar to the virulence genes, the few AMR determinants found in the SAAN isolates are very common in *S. aureus* and have been described for *S. aureus* subsp. *aureus* strains of different origin^40–42^. We observed point mutations in genes that encode for relevant antimicrobial targets and genes encoding for efflux pumps that limit the exposure to antimicrobial agents. Point mutations in antimicrobial target genes can confer a certain degree of resistance. For example, fosfomycin interferes with the function of the UDP-N-acetylglucosamine enolpyruvyl transferase (MurA), which is relevant for cell wall synthesis^43^. A point mutation leading to an amino acid substitution as is described here for *murA* can decrease the affinity for fosfomycin. GlpT is a transporter protein relevant for fosfomycin transport into the bacteria cell. Point mutations in *glpT* therefore affect the cell wall permeability for fosfomycin and act synergistically with *murA* mutations.

MepA, Tet(38), and LmrS are multi-drug efflux pumps belonging to different families and confer cross resistance to different antibiotics and biocides^44^. How far they are expressed in SAAN and biologically active compared to *S. aureus* subsp. *aureus* still has to be determined. At least *mepA* was reported to be impaired by a point-mutation^18^.

The current findings of low genetic diversity obtained with NGS align with other studies that used different molecular techniques^14,18^. This homogeneity may be utilized to develop vaccines that can be broadly applied and reduce the likelihood of pathogen evasion mechanisms. The success of vaccination programs in controlling SAAN in sheep herds was already demonstrated in Spain and the Czech Republic^12,45^ and is currently being investigated by the authors in SAAN-infected small ruminant herds in Germany^46^.

On the farms presented here, carcass condemnations occurred at slaughterhouses due to the presence of abscesses in a significant number of lambs. Therefore, it is important to discuss reports regarding the zoonotic potential of SAAN. Generally, transmission of *S. aureus* from livestock to humans and vice versa occurs, and depending on the lineages of *S. aureus*, the bacteria can cause severe diseases in both animals and humans^47^. A review of the literature reveals only a few reports of SAAN (catalase-negative *S. aureus*) infections in patients from several countries^48–50^. In most described cases, the strains were isolated from patients with a previous medical history. Although the pathogens were identified as catalase-negative *S. aureus*, and SAAN as a causative agent cannot be ruled out, there was no explicit evidence of this pathogen. To our knowledge, the only comprehensive case report of a human with a plausible SAAN infection came from Australia^17^. A farmhand showed severe clinical signs, including alveolar infiltrates, pain, and induration of the abdomen, phlebitis of superficial veins in one leg, and respective inguinal nodes. An obligately anaerobic *S. aureus* was isolated, and epidemiologic circumstances revealed that the man had handled sheep manure without wearing protective gloves. However, MD has not yet been diagnosed in Australian sheep flocks (personal communication Peter Windsor, The University of Sydney, Australia). Due to the limited differentiation of the subspecies *anaerobius* from other *S. aureus* strains, accurately determining the actual occurrence of the pathogen from case reports is challenging. More recent findings suggest a probable human-to-ruminant host transfer in the past and indicate a highly niche-specific ecology of SAAN adapted to sheep and goats^18^. While SAAN infection in patients other than sheep and goats is extremely rare, it cannot be completely ruled out. Considering the diagnostic difficulties, it remains unknown whether SAAN infection might be underdiagnosed in human medicine.

We are aware of the limitations of the present study. The analyzed isolates were collected using a convenience sampling approach and do not represent the prevalence of SAAN in Germany. Details about the probable introduction of SAAN are based on anecdotal reports from farmers due to the lack of surveillance data. Therefore, this information must be interpreted with caution.

In summary, the present study demonstrates for the first time a wide distribution of SAAN in small ruminant herds, especially in southern Germany. Moreover, it closes the epidemiological gap of missing data on SAAN, as noted by others^14^. The trading of infected animals may have facilitated transmission among herds both nationally and internationally. The identified SAAN isolates in small ruminant herds in Germany, Poland, and Austria exhibited low genetic diversity. Although SAAN was first described over 100 years ago, information such as environmental survival rates and zoonotic potential remain vague and should be investigated in future studies.

## Electronic supplementary material

Below is the link to the electronic supplementary material.

Supplementary Material 1

## Data Availability

Data are available upon request from the corresponding author. The assembled genome datasets generated during the current study can be found in the National Center for Biotechnology Information (NCBI) repository, https://www.ncbi.nlm.nih.gov/bioproject, PRJNA634452.
